# Psychotropic drugs in drinking water? A scoping review of evidence on diazepam and fluoxetine

**DOI:** 10.1007/s11356-026-38296-5

**Published:** 2026-10-06

**Authors:** Paula Pinheiro Teixeira, Beatriz Assunção Pereira, Roberta da Silva Carneiro, Lara Júlia Moraes de Assis, Mariana Luiza Leal, Matheus Marcon

**Affiliations:** 1 Laboratório de Estudos Farmacológicos, Instituto de Ciências Biológicas E Naturais, Universidade Federal Do Triângulo Mineiro (UFTM), Praça Manoel Terra, Uberaba, MG 33038025-015 Brazil; 2 Departamento de Bioquímica, Farmacologia E Fisiologia, Instituto de Ciências Biológicas E Naturais, Universidade Federal Do Triângulo Mineiro (UFTM), Uberaba, MG Brazil; 3 Programa de Pós-Graduação Em Ciências da Saúde, Laboratório de Estudos Farmacológicos, Universidade Federal Do Triângulo Mineiro (UFTM), Uberaba, MG Brazil

**Keywords:** Pharmaceuticals, Medications, Emerging contaminants, Drug pollution, Tap water

## Abstract

The global prevalence of mental disorders has increased considerably in recent decades. This evidence, associated with population growth and the expansion of access to medications, favors the growing global consumption of psychotropic drugs. Consequently, an increasing load of these substances is introduced into the environment, especially into water bodies, characterizing them as emerging contaminants. This scoping review study aimed to integrate data and provide a global overview of the occurrence of widely used psychotropic drugs (diazepam, fluoxetine, and haloperidol) in drinking water. The search for studies was conducted across 7 databases, with no language or publication year restrictions. We included only original studies in this review and synthesized data from the included studies for water sampled, sampling location, year of sampling, analytical method used, psychotropic drug found, and concentration found. Our review identified 13 studies published between 2000 and 2024 on the occurrence of diazepam and fluoxetine in tap water, drinking water, bottled water, and drinking well water from 7 countries, with concentrations ranging from 0.2 to 300 ng/L. No studies on the occurrence of haloperidol in drinking water were found. Our data highlight that the occurrence of psychotropic drugs in drinking water is a growing global reality and also show a significant lack of research dedicated to the topic. Furthermore, the results of this scoping review reinforce the need to expand water quality monitoring, improve analytical techniques, invest in advanced water treatment technologies, and implement public policies on the proper disposal of medication and the control of drug pollution.

**Supplementary Information:**

The online version contains supplementary material available at https://doi.org/10.1007/s11356-026-38296-5.

## Introduction

The global prevalence of mental disorders has increased considerably in recent decades, showing a growing trend in the global burden of disease between 1990 and 2019 (GBD 2019 Mental Disorders Collaborators 2022). Additionally, the World Health Organization stated that the COVID-19 pandemic caused an increase of more than 25% in global cases of depression and anxiety worldwide (World Health Organization 2022), thus, the global burden attributed to mental disorders continues to increase (World Health Organization 2022). Currently, the World Health Organization estimates that more than 1 billion people worldwide live with a mental disorder (World Health Organization 2022). This evidence, associated with some factors such as population growth, the inversion of the population’s age structure, the more sensitive recognition and diagnosis of mental illnesses, and the expansion of access to medications, has significantly increased the global consumption of psychotropic drugs (Huang et al. 2025).

A study evaluating pharmaceutical sales data from the IQVIA-Multinational Integrated Data Analysis System reported an average annual increase of 4.08% from 2008 to 2019 (Brauer et al. 2021) in sales of psychotropic medications. In a study in China, data show that the defined daily dose per 1000 inhabitants per day (DDD/1000/day) increased from 4.5 in 2018 to 6.4 in 2021, and total utilization in the hospitals increased by 42.2% (Zhang et al. 2022). In Spain, a study evaluating medications dispensed to primary care patients between 2018 and 2021 reported an upward trend in consumption across both genders and all age groups (García et al. 2023). In Brazil, a study that collected health data from 2015 to 2021 also identified an overall annual increase in sales of psychiatric drugs (Panzenhagen et al. 2025).

This scenario has increasingly favored the introduction of psychotropic drugs into the environment, especially into water bodies, characterizing them as emerging contaminants (Yuan et al. 2024). Psychotropic drugs have been found in various water sources worldwide, such as wastewater (Zergui and Nkontcheu kenko 2024), surface water (Pivetta et al. 2020), groundwater (Silori et al. 2022), freshwater (Fekadu et al. 2019), and even in samples of drinking water (Lv et al. 2019; Muambo et al. 2024). The presence of these substances in the environment is mainly associated with industrial residual discharge, human excretion, since many psychotropic drugs are eliminated in their active form or as active metabolites (Daughton and Ternes 1999; Kümmerer 2009), as well as with the improper disposal of expired or unused medications (Arke et al. 2025; Bound et al. 2006; Bound and Voulvoulis 2005; Seehusen and Edwards 2006). Once in the building’s residual system, the psychotropic drugs present in industrial, domestic, and hospital effluents reach wastewater treatment plants, which are mostly not efficient in completely removing these substances, and end up releasing these molecules into the environment through the discharge of their effluents into the receiving water bodies (Chaber-Jarlachowicz et al. 2025). When in an aquatic environment, psychotropic drugs can disperse throughout watersheds, infiltrate the soil, and reach aquifers, or remain in surface waters used for supply, promoting a recirculation cycle that can reintroduce psychotropic drugs into the drinking water production system (Rodrigues et al. 2025).

Although psychotropic drugs are typically detected at low concentrations in aquatic environments, chronic exposure to these biologically active compounds may induce subtle or long-term effects in aquatic organisms that are not yet fully understood (Formagini et al. 2025). Evidence from the literature reveals several changes at environmentally relevant concentrations, such as behavioral alterations (Chen et al. 2021), changes in growth and reproduction (Ogungbemi et al. 2020; Orozco-Hernández et al. 2022), in the redox state and in activity of the immune system, embryonic and developmental toxicity (Zhu et al. 2023), and bioaccumulation in aquatic species (Weng et al. 2023). Furthermore, human exposure to these emerging contaminants, coupled with the associated health risks, has sparked public interest, particularly with the growing emphasis on the One Health concept, which recognizes human, animal, and environmental health in an integrated way (Atlas 2013; Pettan-Brewer et al. 2024).

The continued presence of psychotropic drugs in the aquatic environment also raises concerns related to bioaccumulation in aquatic organisms and the possible combined effects resulting from simultaneous exposure to multiple chemical compounds. Studies have shown that various drugs and other emerging contaminants can accumulate in the tissues of these organisms and undergo biomagnification along food chains (Aib et al. 2025; Richmond et al. 2018; Sokołowski et al. 2024; Zenker et al. 2014). Additionally, the simultaneous presence of different substances in the environment can result in synergistic or additive effects, even when each compound is present at low concentrations, amplifying potential ecotoxicological impacts and environmental risks (Backhaus and Faust 2012).

Although the presence of drugs in water resources is widely recognized, the occurrence of psychotropic drugs in drinking water is still poorly explored. However, recent evidence indicates the detection of these emerging contaminants in drinking water from different countries. In this context, to fill this gap, this scoping review study aimed to integrate data on the global occurrence of widely used psychotropic drugs in drinking water, aiming to synthesize knowledge on this subject, highlight the importance of public policies and specific regulations for the control of these emerging contaminants, and encourage continuous monitoring and the development of more efficient technological alternatives to remove these contaminants from water.

## Methodology

### Search strategy and identification of studies

This scoping review study followed the Preferred Reporting Items for Systematic Reviews and Meta-Analyses (PRISMA) guidelines extension for Scoping Reviews (PRISMA-ScR) (Tricco et al. 2018). The search for scientific studies was conducted on October 31, 2024, without any language or publication year restriction filters, using 7 databases: CINAHL, Cochrane, Embase, LILACS, PubMed, Scopus, and Web of Science. For the search, restricted and specific search terms were used to suit the characteristics of each database, combining keywords related to psychotropic drugs (“diazepam” OR “fluoxetine” OR “haloperidol”) AND drinking water (“bottled water” OR “drinking water” OR “potable water” OR “tap water”) and their descriptors, together with the Boolean operators "OR" and "AND". This scoping review was registered in Open Science Framework, and the complete search strategies employed in each database are available in https://osf.io/6w3pc/overview.

### Eligibility screening

The articles identified from the databases were transferred into the Rayyan® web platform, which was used throughout the study selection process. Initially, duplicate studies were identified using the tool for identification available in Rayyan (Ouzzani et al. 2016). Subsequently, studies identified as duplicates were manually evaluated and excluded after confirmation. After excluding duplicates, studies were selected based on predefined inclusion/exclusion criteria. This analysis was performed by two independent researchers (B.A.P. and M.L.L.) who rigorously assessed the studies for eligibility. Initially, studies were selected based on the title/abstract, and when this was not possible, we proceeded to analyze the full text. Discrepancies between two researchers were resolved by a third reviewer (P.P.T.). After completing the selection process, all references from the included studies were reviewed to identify studies that may not have been found during the database search.

Studies were included in this review when they met the following criteria: (1) original studies; (2) reported the occurrence of diazepam, fluoxetine, and haloperidol in drinking water. Studies were excluded from this review when they met the following criteria: (1) studies of the type review, letter, editorial, commentary, conference proceedings, corrections, book chapter, abstract, or any other type of publication that did not correspond to original data; (2) studies that did not analyze the presence of diazepam, fluoxetine, or haloperidol in drinking water (e.g., experimental studies in animals) or studies that used analytical methods of detection by bioassay or polar organic chemical integrative sampler (POCIS); (3) studies that did not find diazepam, fluoxetine, or haloperidol in the sample analyzed; (4) studies that analyzed the presence of diazepam, fluoxetine, or haloperidol in samples that were not drinking water or that enriched the drinking water sample with the drug; (5) studies that reported the analytical result as equal to or below the limit of quantification of the method (< LOQ); and (6) studies that were not available in full.

### Data extraction

The extraction of data was performed by three independent researchers (B.A.P., R.S.C., and L.J.M.A) based on analysis of the full text, figures, and supplementary material from the studies included in this review. The following information was collected: (1) metadata (study ID (title, author, and year of publication), water sampled, sampling location, year of sampling, analytical method used); (2) psychotropic drug found; (3) concentration found. Data on the concentrations reported in the included studies were primarily extracted from the maximum concentration. In this review, we present all concentrations in nanograms per liter (ng/L) to facilitate comparison between studies. The graphic elements of this study were created using Microsoft Office 365 (version 16.106.1).

## Results

### Studies selected

**Fig. 1:**
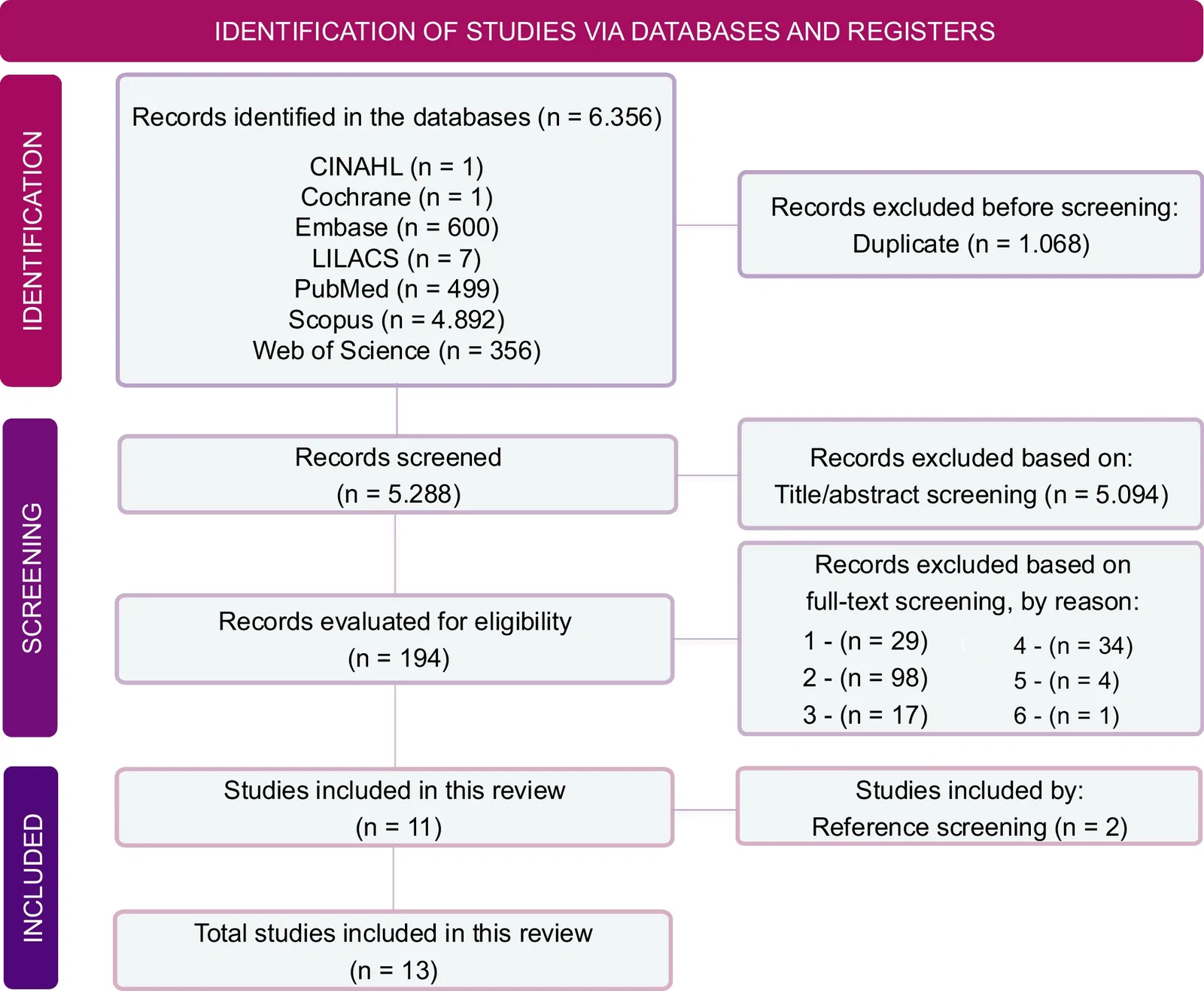
Flowchart of the study screening and selection process. The diagram details the database searches, the number of records screened, and the number of studies included in the review. The complete exclusion reasons can be found in the methodology section

We identified 6.356 studies across seven consulted databases: CINAHL (1), Cochrane (1), Embase (600), LILACS (7), PubMed (499), Scopus (4.892), and Web of Science (356). After excluding 1.068 duplicate records, 5.288 records remained for screening. Subsequently, 5.094 records were excluded by the title/abstract screening. Then, 194 records were assessed for eligibility, and after full-text screening, a further 182 records were excluded for the following reasons: 1 (*n* = 29); 2 (*n* = 98); 3 (*n* = 17); 4 (*n* = 34); 5 (*n* = 3); 6 (*n* = 1). Finally, 11 studies met the inclusion/exclusion predefined criteria for this review. Additionally, after screening the references of these included studies, two additional records were identified that met the eligibility criteria and were also included in this review (Benotti et al. 2009; Zuccato et al. 2000). Therefore, as shown in Fig. 1, 13 studies were included in this review. The complete list of included articles is described in Table 1 and can be found at https://osf.io/6w3pc/overview.

**Table 1:** Studies that reported diazepam and fluoxetine in drinking water worldwide

Drug	Conc. (ng/L)	Sample Water	Sampling Location	Analytical Method	Year	Study ID
Diazepam	23.5 0.2	Drinking water	Lodi, Italy Varese, Italy	HPLC–MS/MS	Unclear	(Zuccato et al. 2000)
	0.33	Drinking water	USA	LC–MS/MS GC–MS/MS	2006 2007	(Benotti et al. 2009)
	3.2	Drinking water	Shanghai, China	LC–MS/MS	2014	(Wu et al. 2015)
	1.22	Tap water	Beijing, China	UPLC-MS/MS	2017	(Zhang et al. 2019)
	24.91	Tap water	China	UPLC-ESI-QqQ/MS	2018 2019	(Wang et al. 2020)
	0.25	Drinking well water	Budapest, Hungary	SFC-MS/MS	2017 2018	(Kondor et al. 2020)
	22.4	Tap water	Eastern China	UPLC-ESI-QqQ/MS	2018	(Wang et al. 2024)
	0.33	Drinking water	South Korea	UPLC-MS/MS	2022	(Muambo et al. 2024)
Fluoxetine	0.82 0.64	Drinking water Tap water	USA	LC–MS/MS GC–MS/MS	2006 2007	(Benotti et al. 2009)
	200–300 200–300	Tap water Bottled water	New Jersey, USA	UHPLC-LTQ- Orbitrap	Unclear	(Pinhancos et al. 2011)
	19.2	Drinking water	USA	HPLC–MS/MS	2009 2010	(Padhye et al. 2014)
	0.2	Tap water	Shanghai, China	LC–MS/MS	2014	(Wu et al. 2015)
	0.27	Drinking water	East Anglia, England	UPLC-MS	Unclear	(Peng et al. 2019)
	2.08 2.08	Drinking water Tap water	China	UPLC-MS/MS	2016	(Lv et al. 2019)
	164	Bottled water	Bogotá, Colombia	GC–MS	Unclear	(Enríquez-Hidalgo et al. 2022)

*CONC*, Concentration; *GC–MS*, Gas Chromatography–Mass Spectrometry; *GC–MS/MS*, Gas Chromatography–Tandem Mass Spectrometry; *HPLC–MS/MS*, High-Performance Liquid Chromatography–Tandem Mass Spectrometry; *LC–MS/MS*, Liquid Chromatography–Tandem Mass Spectrometry; *SFC-MS/MS*, Supercritical Fluid Chromatography–Tandem Mass Spectrometry; *UHPLC-LTQ-Orbitrap*, Ultra-High-Performance Liquid Chromatography–Linear Trap Quadrupole–Orbitrap Mass Spectrometry; *UPLC-ESI-QqQ/MS*, Ultra-Performance Liquid Chromatography–Electrospray Ionization–Triple Quadrupole Mass Spectrometry; *UPLC-MS*, Ultra-Performance Liquid Chromatography–Mass Spectrometry; *UPLC-MS/MS*, Ultra-Performance Liquid Chromatography–Tandem Mass Spectrometry; *USA*, United States of America

### Diazepam and fluoxetine in drinking water worldwide

All the 13 studies included in this review were published between 2000 and 2024. In total, 8 studies reported the occurrence of diazepam, and 7 reported the occurrence of fluoxetine in drinking water. No studies on the occurrence of haloperidol in drinking water were found (Table 1).

**Fig. 2:**
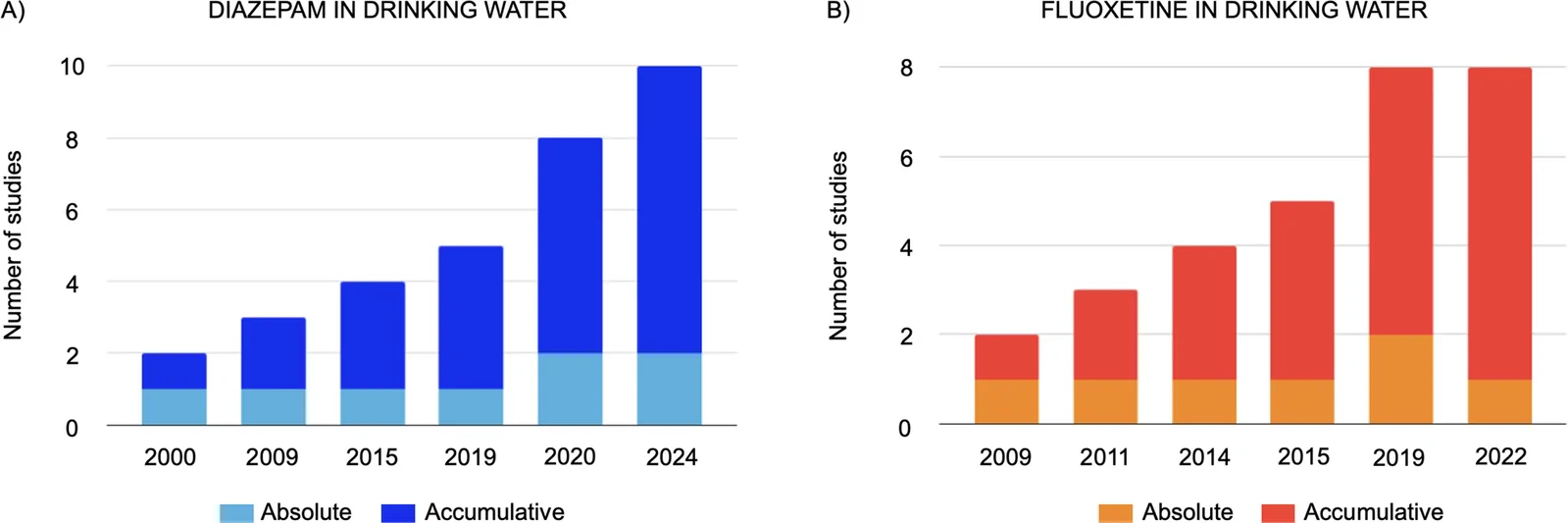
Absolute and accumulative number of studies published on the occurrence of diazepam (**A**) and fluoxetine (**B**) in drinking water worldwide

The absolute and cumulative number of studies on diazepam and fluoxetine in drinking water are shown in Fig. 2A and B, respectively. The first study on the occurrence of diazepam in drinking water was published in 2000 and reported concentrations of 23.5 and 0.2 ng/L in the cities of Lodi and Varese, Italy, respectively. Over the years, only 7 other publications reported the occurrence of diazepam in drinking water. In 2020 and 2024, the largest number of publications per year occurred, with two each (Fig. 2A). On the other hand, the first study on the occurrence of fluoxetine in drinking water was published in 2009, reporting concentrations of 0.82 and 0.64 ng/L in drinking water and tap water, respectively, in the United States of America (USA). After that date, only 6 other studies were published, with the largest number of publications per year occurring in 2019, with two studies published (Fig. 2B).

### Geographical distribution of diazepam and fluoxetine in drinking water worldwide

**Fig. 3:**
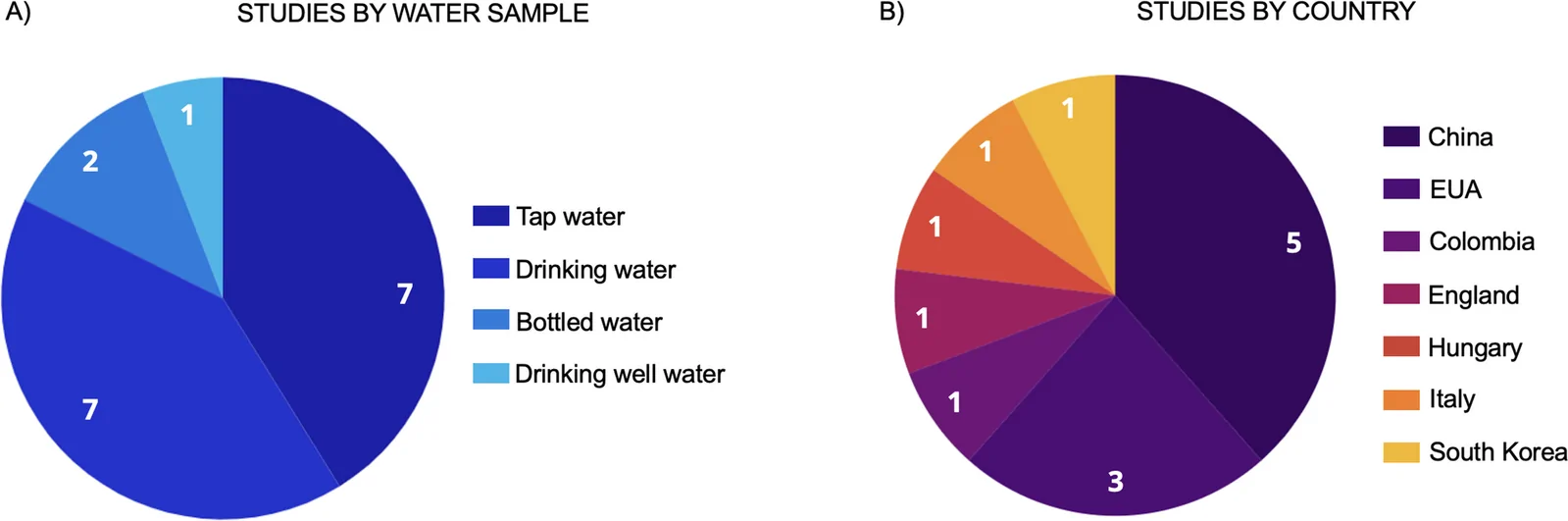
The number of studies concerning the occurrence of diazepam and fluoxetine in drinking water worldwide by water sample (**A**) and by country (**B**)

The reviewed studies showed diversity in the water bodies analyzed, in the geographic locations of sampling, and in the range of concentrations reported for diazepam and fluoxetine in drinking water. The occurrence of diazepam and fluoxetine was reported in samples of tap water (*n* = 7; 53.84%), drinking water (*n* = 7; 53.84%), bottled water (*n* = 2; 15.38%), and drinking well water (*n* = 1; 7.69%) (Fig. 3A) of 7 countries of Asia, America and Europe. The studies were predominantly from China (*n* = 5; 38.46%) and the USA (*n* = 3; 23.08%), followed from Colombia (*n* = 1; 7.69%), England (*n* = 1; 7.69%), Hungary (*n* = 1; 7.69%), Italy (*n* = 1; 7.69%), and South Korea (*n* = 1; 7.69%) (Fig. 3B).

**Fig. 4:**
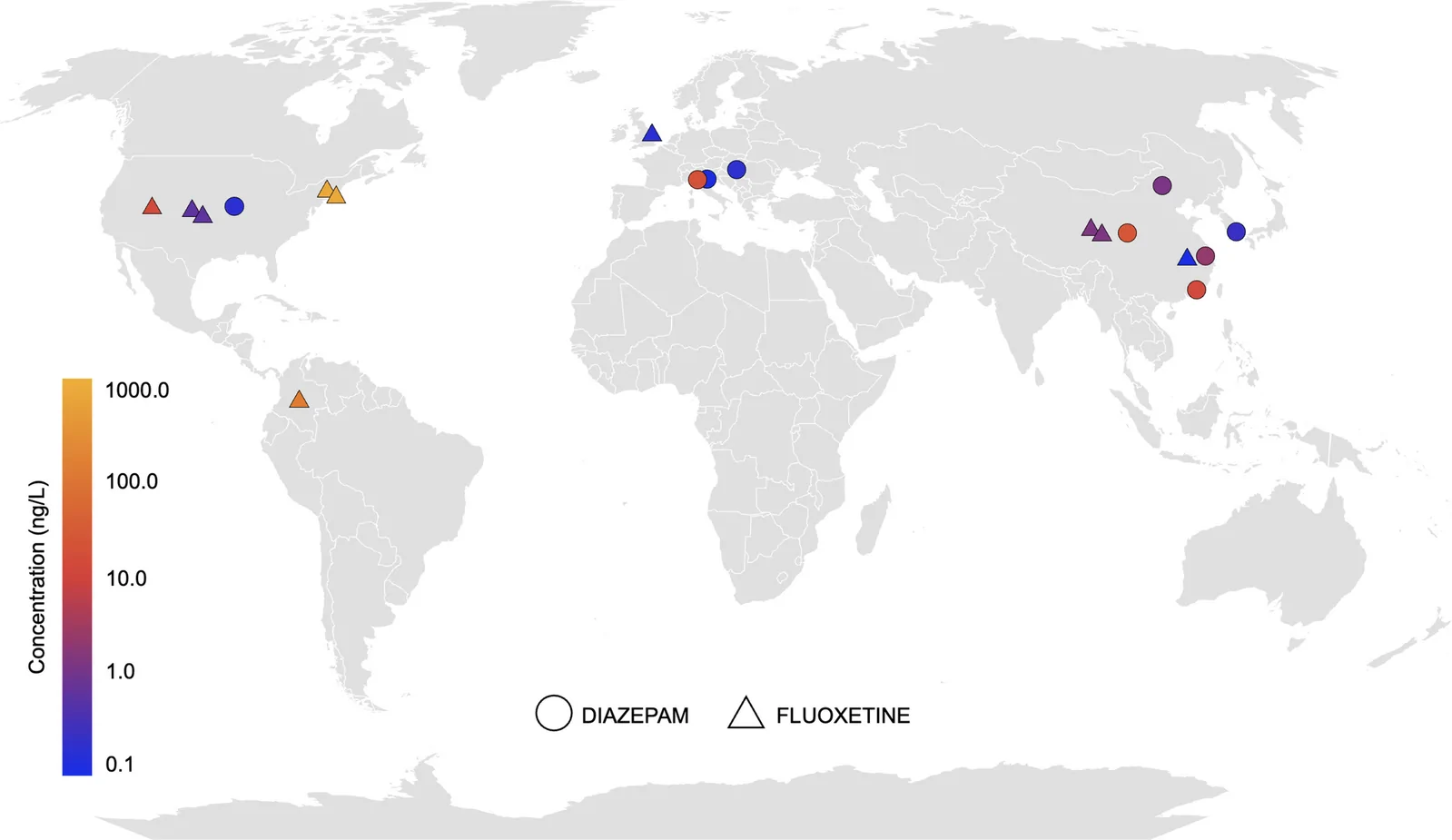
Overview of the global occurrence of diazepam and fluoxetine in drinking water. The concentration of psychotropic drugs at each location is represented as the maximum concentration reported in the studies included in this review

The diazepam concentration found in the analyzed samples ranged from 0.2 to 24.91 ng/L, while the fluoxetine concentration found in the analyzed samples ranged from 0.2 to 300 ng/L (Fig. 4 and Table 1). The global geographic distribution of diazepam and fluoxetine occurrence in drinking water and the concentrations found at each detection location are shown in Fig. 4.

## Discussion

### General

Our review identified 13 studies published between 2000 and 2024 reporting diazepam and fluoxetine in the drinking water of 7 countries. No studies on the occurrence of haloperidol in drinking water were found. Research on the occurrence of psychotropic drugs in drinking water is still very recent, since the first publications specifically on diazepam and fluoxetine in drinking water appeared in the early 2000s. Furthermore, the number of studies published annually remains very low, consumption of these substances continues to rise, and most conventional water treatment systems currently in operation lack the capacity to eliminate them. The absence of published studies on haloperidol should not be interpreted as evidence that the compound is absent from drinking water. Instead, it indicates a current lack of monitoring data and research attention. This lack of studies on monitoring these substances in drinking water becomes even more evident when we consider the recent methodological advances in techniques for monitoring emerging contaminants in aquatic environments (Paszkiewicz et al. 2022; Richardson and Ternes 2022).

### Diazepam and fluoxetine in drinking water worldwide

The results of this review revealed that diazepam and fluoxetine have been found in drinking water at concentrations ranging from 0.2 to 300 ng/L. Of the 13 studies included in this review, 8 reported the occurrence of diazepam, and 7 reported the occurrence of fluoxetine in drinking water. Tap water and bottled water were the water samples in which diazepam and fluoxetine were reported in the highest concentrations. Diazepam was detected at the highest concentration (24.91 ng/L) in tap water (Wang et al. 2020) and at the lowest concentration (0.2 ng/L) in drinking water (Zuccato et al. 2000), while fluoxetine was detected in both tap water and bottled water at the highest concentration (300 ng/L) (Pinhancos et al. 2011), and at the lowest concentration (0.2 ng/L) in tap water.

The studies analyzed revealed the presence of diazepam and fluoxetine in the drinking water of 7 countries, with the largest number of publications from samples collected in developed countries, such as China (*n* = 5) and the USA (*n* = 3). The predominance of studies in these developed countries likely reflects the high scientific, technological, and financial capacity, which concentrates a large share of global investment in research, advanced laboratory infrastructure, and large-scale environmental monitoring programs. Furthermore, both have intense industrial activity and high consumption of medications, which naturally drives scientific interest in investigating the occurrence and environmental impacts of psychotropic drugs in drinking water. On the other hand, these data also reveal a global asymmetry in the production of scientific knowledge about psychotropic drugs as drinking water contaminants, since in developing countries, the consumption of psychotropic drugs is increasing, but the capacity for environmental monitoring is still limited. Therefore, we believe this demonstrates an important gap in understanding the actual distribution of these contaminants in drinking water worldwide, since other regions with limited resources, funding, and scientific infrastructure may remain underrepresented in the scientific literature.

### How do psychotropic drugs get into drinking water?

The occurrence of psychotropic drugs in drinking water is associated with several factors, such as the ever-increasing production and consumption of these substances, inadequate human disposal, and the limitations of conventional water treatment systems. Furthermore, although there are still few published studies on the subject, the increased detection capacity in recent years, driven by methodological advances, has contributed to the identification of increasingly lower concentrations of psychotropic drugs in the environment (Organisation for Economic Co-operation and Development 2019).

Pharmaceutical manufacturing has increasingly been recognized as a significant and often underestimated source of environmental contamination, particularly in regions with high industrial activity and insufficient regulatory oversight. During the production process, active pharmaceutical ingredients and their intermediates may be released into wastewater streams at concentrations substantially higher than those originating from domestic use, leading to localized contamination hotspots (aus der Beek et al. 2016; Cardoso et al. 2014; Larsson 2014). Studies have reported extremely elevated levels of drugs in effluents from manufacturing facilities, sometimes exceeding those detected in municipal wastewater by several orders of magnitude, raising concerns about drugs as emerging contaminants (Adetunde et al. 2025; Phillips et al. 2010). Consequently, pharmaceutical production sites represent critical point sources of contamination in water matrices, contributing to the persistence and dissemination of bioactive compounds with potential long-term impacts on aquatic ecosystems and environmental and human health.

The consumption of psychotropic drugs also contributes as an important route of contamination. After administration and absorption by the body, drugs undergo metabolic processes that can result in partial or total transformation of the ingested substance. However, in the case of psychotropic drugs, a considerable fraction is usually excreted from the body, mainly via urine, in unchanged form or as active metabolites, which reach the residential and urban wastewater systems and, finally, wastewater treatment plants (Jelic et al. 2011). In general, the administered dose of a drug is rarely completely degraded by the body, and a significant portion is eliminated mainly through urine (Winker et al. 2008). Therefore, the excretion of psychotropic drugs by the body contributes as an important source of these substances entering effluents, and, consequently, in water matrices.

Improper disposal of medications, whether directly into sinks, toilets, or household waste, is still practiced both in homes and healthcare institutions (Organisation for Economic Co-operation and Development 2022). To mitigate this problem, programs for collecting expired or unused medications have been implemented, providing safe, environmentally sound disposal. Such initiatives, known as take-back programs, represent an effective strategy to prevent the contamination of surface waters and supply systems by drugs (Kinrys et al. 2018). However, data from some studies highlighted that this is still a common problem. For example, the Canadian government’s national statistics agency shows that approximately 39% of Canadian families disposed of medications improperly, such as by burying, flushing, or including them in the garbage (Government of Canada 2008). A study conducted with medication consumers in Brazil reports that 76% of them dispose of unused medications in the regular trash, down the toilet, or in the sink (Lago et al. 2022). Another study conducted in China with 6293 families reported that 2771 (44%) had unwanted medications at home, and the majority (2465, 89%) of these families disposed of them in the trash, sink, or toilet (Yang et al. 2025). In Saudi Arabia, a cross-sectional study revealed that 73% of respondents throw their medications in the trash, 14% return them to a pharmacy, 5% never discard them, and 3% donate them to a friend or charity (AlAzmi et al. 2017).

Once in the environment, psychotropic drugs can reach drinking water. The main contamination pathway occurs through infiltration into the soil, access to groundwater, and transport to surface waters (rivers, lakes, and springs), and/or for supply (Chia et al. 2025; Gworek et al. 2021). On the other hand, when in urban effluents, psychotropic drugs reach wastewater treatment plants that are currently ineffective at completely removing these substances, and do not prevent the total dispersion of psychotropic drugs into surface waters (e.g., rivers, streams, lakes, lagoons, sea, dams, and reservoirs) used for supply and/or drinking water production systems (Mazhandu and Mashifana 2024).

### Limitations of drinking water treatment plants in removing psychotropic drugs

Conventional drinking water treatment plants are designed primarily to remove pathogens and have limitations in efficiently eliminating organic micropollutants, such as psychotropic drugs, from water (Coimbra et al. 2021). The physicochemical properties of these substances make them chemically stable and resistant to conventional water treatment processes, such as coagulation, flocculation, sedimentation, sand filtration, and chlorination (Grandclément et al. 2017). Therefore, psychotropic drugs and their metabolites remain in the effluents of conventional drinking water treatment plants (Zanni et al. 2024). Since the psychotropic drugs were designed to be active at low concentrations, they can cause effects in non-target organisms. Therefore, psychotropic drugs are considered persistent organic pollutants or emerging contaminants (Patel et al. 2019).

Some studies conducted on drinking water treatment plants demonstrate variable removal efficiencies depending on the compound and treatment configuration, highlighting that conventional processes alone are insufficient and that advanced treatments such as ozonation, activated carbon, membranes, or advanced oxidation processes are often required to achieve satisfactory removal (Boleda et al. 2011; Grancha et al. 2025).

In this context, the persistent detection of psychotropic drugs in treated drinking water is an extremely important strategy to understand the dimension of the water treatment problem and to reflect the inherent technological and operational limitations of conventional treatment infrastructure, emphasizing the urgent need for the implementation of advanced treatment technologies and comprehensive monitoring frameworks to mitigate potential environmental and public health risks.

### Future perspectives and research gaps

The growing global awareness of water contamination signals concerns for global public health, considering its far-reaching impacts on the entire biosphere. The United Nations emphasizes the need for intersectoral and multidisciplinary measures, with an integrated vision of healthcare, considered essential for achieving the Sustainable Development Goals of the 2030 Agenda, which consists of a global action plan composed of objectives and targets that address the economic, social, and environmental dimensions in an integrated manner among countries (United Nations 2015). In this context, the term One Health has been scientifically recognized and consolidated for its social relevance since 2008, integrating the vision of human, animal, and environmental health (Pitt and Gunn 2024).

Despite some advances, we believe that even more robust and coordinated efforts are essential, since the complexity of this problem becomes still more evident when viewed from a global perspective, considering the diversity of pharmacological classes consumed daily, the continuous introduction of new compounds into the environment, the transformation products, and the toxicity of mixtures. Furthermore, the forecast for the consumption of psychotropic drugs and other medications indicates substantial increases in sales, pointing to an even greater load of emerging contaminants in water matrices (IQVIA 2024).

Although we know about the occurrence of psychotropic drugs in drinking water, this is still just the tip of the iceberg. There are significant gaps in knowledge regarding the extent, impacts, and management of this issue. Future research should focus on improving analytical monitoring and prioritizing scientific investment in underrepresented regions to achieve more geographically balanced and globally representative drinking water assessments. In addition, advances in drinking water treatment technologies are urgently needed to ensure effective removal of these substances from drinking water and mitigate risks. Educational strategies promoting the rational use of medications and proper disposal practices should also be strengthened to reduce environmental contamination. However, such solutions still face significant barriers, including high costs and the need for specialized infrastructure, which limits their large-scale implementation, especially in developing countries (Kumar et al. 2026). Furthermore, the potential human health risks associated with chronic exposure to psychotropic drugs in drinking water remain largely unexplored and require in-depth investigation.

## Conclusion

This scoping review highlighted that the occurrence of psychotropic drugs in drinking water is a growing global reality, driven by several factors, such as ever-increasing production and consumption of these substances, inadequate human disposal, and the limitations of conventional water treatment systems. We found 13 studies on the occurrence of diazepam and fluoxetine in drinking water from 7 countries in Asia, America, and Europe, in concentrations ranging from 0.2–24.91 and 0.2–300 ng/L, respectively. No studies on the occurrence of haloperidol were found. However, the absence of published studies on haloperidol should not be interpreted as evidence that the compound is absent from drinking water. Instead, it indicates a current lack of monitoring data and attention to research. Although the occurrence of psychotropic drugs in drinking water is a reality, there is a significant lack of research dedicated to the topic, since the literature still lacks studies. Therefore, given the importance of this issue for global health, monitoring studies worldwide are necessary to obtain a more balanced and globally representative environmental assessment. Furthermore, the results of this scoping review reinforce the need to expand environmental surveillance, improve analytical techniques, invest in advanced treatment technologies, and strengthen public policies aimed at the proper disposal of medications and the control of drug pollution.

## Supplementary Information

Below is the link to the electronic supplementary material.

Supplementary file1 (PDF 99 KB)

## Data Availability

All relevant data are included in the paper, and supporting data are available in the Open Science Framework (https://osf.io/6w3pc/overview).
